# The Role of Vitamin D in Severity and Control of Asthma in Children and Adolescents: A Systematic Review and Meta‐Analysis

**DOI:** 10.1002/ppul.71541

**Published:** 2026-02-27

**Authors:** Joelia M. Ladeira, Olívia Zacas, Amanda Miranda Ferreira, Patrícia Chaib Gomes Stegun, Milena Baptistella Grotta, Adyleia A. D. Contrera Toro

**Affiliations:** ^1^ Department of Pediatric State University of Campinas (UNICAMP) Campinas São Paulo Brazil

**Keywords:** 25 hydroxyvitamin D, IgE, lung diseases, T cells

## Abstract

**Context:**

Vitamin D may modulate the inflammatory processes involved in asthma.

**Objective:**

To synthesize evidence on the association between serum 25‐hydroxyvitamin D levels and asthma control, severity, pulmonary function, and inflammatory markers in children and adolescents.

**Data Sources:**

MEDLINE PubMed, BIREME, EBSCOhost, Scopus, Web of Science, EMBASE, Cochrane Library, and ProQuest until May 2025.

**Study Selection:**

Eligible studies enrolled participants aged 2–18 years with clinically diagnosed asthma and evaluated serum vitamin D levels in relation to asthma severity and/or control and pulmonary function parameters or type 2 inflammatory biomarkers. We included observational and randomized studies.

**Data Extraction:**

The risk of bias for studies was assessed. Random‐effects models estimated pooled outcomes. The heterogeneity was assessed using Cochran's Q and the I² statistic.

**Results:**

Forty‐one studies (7780 participants) were included; 25 contributed to meta‐analyses. Children with asthma had significantly lower serum 25(OH)D levels than healthy controls (mean difference −4.89 ng/mL; 95% CI −7.38 to −2.40; *p* < 0.001). Severe asthma was associated with lower vitamin D compared with mild disease (−4.21 ng/mL; 95% CI −6.43 to −1.98; *p* = 0.0002). No significant difference was observed between controlled and uncontrolled asthma. Correlations with pulmonary function were weak and non‐significant (FEV₁ *r* = 0.18; *p* = 0.08). Vitamin D showed a moderate inverse association with total IgE (*r* = −0.37; *p* = 0.02), but not with eosinophil counts or IL‐10. Heterogeneity was high across analyses.

**Conclusions:**

Children with asthma exhibit lower serum vitamin D levels compared with healthy peers, and these levels are inversely associated with asthma severity and total IgE. No consistent associations were observed with pulmonary function or asthma control. Further research is needed to determine whether correcting vitamin D deficiency can improve clinical and immunologic outcomes in pediatric asthma.

**Abbreviations**
IL4: Interleukin 4
IL5: Interleukin 5
IL10: Interleukin 10
JBI: Joanna Briggs Institute
NOS: Newcastle–Ottawa scale
PICOS: Population, Intervention, Comparison, Outcomes, Study Design
PRISMA‐P: Preferred Reporting Items for Systematic Review and Meta‐Analysis
PROSPERO: International Prospective Register of Systematic Reviews Protocols
RoB: Risk of Bias in Randomized Trials
TGF‐β: Transforming Growth Factor Beta
Th1: Helper T lymphocytes 1
Th17: Helper T lymphocytes 17
Th2: Helper T lymphocytes 2
Treg T: Regulatory T lymphocytes

## 1 Introduction

In recent years, vitamin D has attracted increasing attention as a potential immunomodulatory factor in the pathophysiology of asthma [1]. Beyond its classical role in calcium homeostasis and bone health, vitamin D influences both innate and adaptive immune responses by modulating T‐helper cell differentiation, cytokine production, and the expression of vitamin D receptors (VDRs) in airway epithelial and immune cells [1, 2]. Through these mechanisms, adequate serum concentrations of 25‐hydroxyvitamin D [25(OH)D] may attenuate airway inflammation, improve lung function, and reduce the frequency of asthma exacerbations, although findings across studies remain inconsistent [3, 4].

Observational studies have consistently demonstrated that vitamin D deficiency is highly prevalent among children with asthma and has been associated with increased disease severity, poorer symptom control, and impaired pulmonary function, even in regions with abundant sunlight [5, 6]. Moreover, systematic reviews and meta‐analyses suggest that vitamin D supplementation may reduce the risk of severe exacerbations and hospitalizations, although its overall impact on disease control remains controversial [4, 7, 8].

Beyond its potential clinical effects, vitamin D has been shown to modulate several inflammatory biomarkers relevant to asthma pathogenesis, including total IgE, eosinophil counts, and cytokines such as IL‐4, IL‐5, and IL‐13 [2, 9]. Experimental studies also indicate that vitamin D may influence airway remodeling through effects on epithelial integrity and smooth muscle proliferation, further supporting its possible therapeutic role in asthma [9].

Despite these observations, considerable variability remains among published studies due to differences in population characteristics, vitamin D measurement assays, study design, and definitions of asthma severity and control. As a result, the relationship between vitamin D status, airway inflammation, and clinical outcomes in pediatric asthma remains incompletely understood.

Therefore, the objective of this systematic review and meta‐analysis is to synthesize the available evidence on the association between serum vitamin D levels and asthma control and severity in children and adolescents, and to evaluate their correlation with pulmonary function and inflammatory biomarkers.

## 2 Methods

### 2.1 The Registration

This systematic review protocol was registered with the International Prospective Register of Systematic Reviews (PROSPERO) [10] in January 2021 (registration number CRD42021221638). This protocol was developed in accordance with the Preferred Reporting Items for Systematic Review and Meta‐Analysis Protocols (PRISMA‐P) 2020 statement guidelines [11]. Furthermore, the guiding question was elaborated to ensure the systematic search of scientific literature using PICOS (Population/Intervention/Comparison/Outcomes/Study Design).

### 2.2 Search Strategy

This systematic review was based on articles published until May 2025. We searched the following electronic bibliographic databases: PubMed, BVS‐BIREME, Embase, EBSCOhost, Scopus, Web of Science, ProQuest, and the Cochrane Library and used the following search strategy: Children OR Child, Adolescent OR Adolescents OR Adolescence OR Teens OR Teen OR Teenagers OR Teenager OR Youth OR Youths OR “Adolescents, Female” OR “Adolescent, Female” OR “Female Adolescent” OR “Female Adolescents” OR “Adolescents, Male” OR “Adolescent, Male” OR “Male Adolescent” OR “Male Adolescents AND Asthma OR Asthmas OR “Bronchial Asthma” OR “Asthma, Bronchial” AND “Vitamin D” Cholecalciferol OR “Vitamin D 3” OR “Vitamin D3” OR Cholecalciferols OR Cholecalciferol AND “Inflammation Mediators” OR Lymphocytes OR Biomarkers OR Interleukins. Literature screening was conducted using Rayyan [12], a web‐based platform that facilitated collaboration among reviewers during the screening process. Screening was conducted in two stages: initially by title and abstract, followed by full‐text review. In cases of unresolved disagreement, a third reviewer was consulted. Duplicate articles were identified and excluded.

### 2.3 Data Extraction and Management

Data from eligible articles were independently extracted by 2 reviewers (J.L. and O.Z) and any disagreement was resolved by discussion by a third reviewer (M.G.). The extracted data were as follows: authors, year of publication, country of study, study types, number of participants, asthma diagnostic methods, results and outcome. When essential data were missing, we contacted the corresponding authors of the study to get the missing data.

### 2.4 Inclusion and Exclusion Criteria

Studies including children and adolescents aged 2 to 18 years, regardless of sex and ethnicity, were considered eligible, provided that asthma was diagnosed according to clearly defined and internationally recognized criteria. Articles assessing vitamin D levels in relation to asthma control and severity, as well as their association with type 2 (T2) inflammation and lung function, were included. The outcomes of interest comprised: (1) the relationship between vitamin D levels and asthma control and severity, (2) the correlation between serum vitamin D levels and lung function, and (3) the correlation of vitamin D in T2 inflammation. Eligible study designs included primary research, prospective observational studies, and randomized controlled trials, published in English. Exclusion criteria were case reports, letters to the editor, studies involving adults, children younger than 2 years, participants with comorbidities, pregnant women, elderly populations, or animal studies.

### 2.5 Risk of Bias Assessment

The risk of bias for studies was assessed using the tools the Joanna Briggs Institute (JBI) Critical Assessment Scale [13] for cross‐sectional studies, the Newcastle‐Ottawa scale (NOS) [14] for case‐control and cohort studies and Cochrane risk of bias tool, RoB 2 for the studies RCTs [15].

### 2.6 Statistical Methods

Meta‐analyses were performed using R software (version 4.3.3) with the “meta” package [16].

For continuous outcomes, pooled mean differences and 95% confidence intervals were estimated using a random‐effects model via the metacont function. Heterogeneity was assessed using Cochran's Q test and quantified by I² statistics [17].

Results were presented in forest plots. For correlation analyses between vitamin D levels, inflammatory markers, and lung function, correlation coefficients were transformed to Fisher's Z scores and pooled using the metagen function. Forest plots displayed both transformed and original correlation values. Publication bias was evaluated using funnel plots and Egger's regression test [18].

A two‐sided *p*‐value < 0.05 was considered statistically significant.

## 3 Results

**Figure 1:**
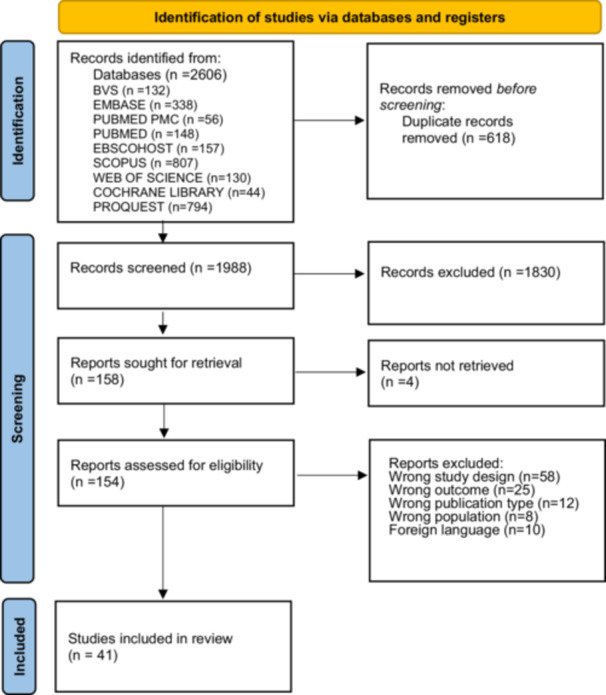
PRISMA 2020 (preferred reporting items for systematic and meta‐analysis). [Color figure can be viewed at wileyonlinelibrary.com]

During the review process and literature search, we identified 2606 manuscripts (Figure 1) from an initial search of the databases. After removing duplicates, 1988 citations remained. Of these, 1630 were excluded after evaluating the titles/abstracts, as their contents did not fit within the inclusion criteria of this review. Based on full‐text screening, we agreed on 41 articles eligible for this review.

### 3.1 Characteristics of the Included Studies

A total of 41 studies met the inclusion criteria (Table 1), comprising 19 cross‐sectional studies [19, 20, 21, 22, 23, 24, 25, 26, 27, 28, 29, 30, 31, 32, 33, 34, 35, 36, 37], three cohort studies [5, 38, 39], 13 case‐control studies [3, 40, 41, 42, 43, 44, 45, 46, 47, 48, 49, 50, 51], and six randomized controlled trials [52, 53, 54, 55, 56, 57]. The publication period was until May 2025, with a total of 7.780 participants. The meta‐analysis was carried out in 25 studies. The study population consisted of children between the ages of two and eighteen with a confirmed diagnosis of asthma who receive care at specialized centers in the city and country of origin of the studies.

**Table 1:** The characteristics of included studies.

Author	Country	Study design	Number of patients	Age (years)
Aldubi et al. [19]	Saudi Arabia	cross‐sectional	70	4–18
Amorim et al. [20]	Brazil	cross‐sectional	26	6–12
Asseri et al. [21]	Saudi Arabia	cross‐sectional	432	3–12
Arikoglu et al. [22]	USA	cross‐sectional	67	10.0 (3.7)*
Aziz et al. [23]	Pakistan	cross‐sectional	114	6–18
Brehm et al. [24]	USA	cross‐sectional	616	7–10
Brehm et al. [25]	Puerto Rico	cross‐sectional	560	6–14
Chinellato et al. [26]	Italy	cross‐sectional	75	5–11
Dabbah et al. [27]	Israel	cross‐sectional	71	6–18
Han et al. [28]	Puerto Rico	cross‐sectional	523	6–14
Kaur et al. [29]	India	cross‐sectional	100	5–15
Krobtrakulchai et al. [30]	Thailand	cross‐sectional	125	6–18
Kuti [31]	Nigeria	cross‐sectional	180	2–15
Omole et al. [32]	Nigeria	cross‐sectional	206	2–14
Suprun et al. [33]	Russia	cross‐sectional	167	2–14
Srivastava et al. [34]	India	cross‐sectional	175	2–15
Szentpetery et al. [35]	Puerto Rico	cross‐sectional	578	6–14
Tanega‐Aliling [36]	Philippines	cross‐sectional	44	3–18
Uysalol et al. [37]	Turkey	cross‐sectional	170	2–14
Malheiro et al. [5]	Brazil	cohort	141	7–17
Batmaz et al. [38]	Turkey	cohort	30	7–17
Wu et al. [39]	USA	cohort	1024	5–12
Chary et al. [3]	India	case control	120	2–6
Abushouk et al. [40]	Saudi Arabia	case control	95	6–14
Bai et al. [41]	China	case control	246	8.93 (2.73)*
Dogru et al. [42]	Turkey	case control	194	3–8.5
Maalmi et al. [43]	Tunisia	case control	69	6–16
Ismail et al. [44]	Egypt	case control	72	3–14
Kalick et al. [45]	Poland	case control	52	5–8
Kalmarzi et al. [46]	Kurdistan Province, Iran	case control	120	6–18
Mohammadzadeh et al. [47]	Iran	case control	200	5–14
Pervaiz et al. [48]	Pakistan	case control	62	5–14
Singh et al. [49]	India	case control	300	5–15
Wang et al. [50]	China	case control	90	4.3 (1.4)*
Wawrzyniak et al. [51]	Poland	case control	40	5–12
Bar et al. [52]	Israel	RCT	39	6–18
Kerley et al. [53]	Ireland	RCT	44	6–16
Rosser et al. [54]	USA	RCT	174	6–16
Tachimoto et al. [55]	Japan	RCT	89	6–15
Thakur et al. [56]	India	RCT	110	6–11
Wu et al. [57]	China	RCT	170	6–13

Abbreviation: NR, not reported.

* Mean.

**Figure 2:**
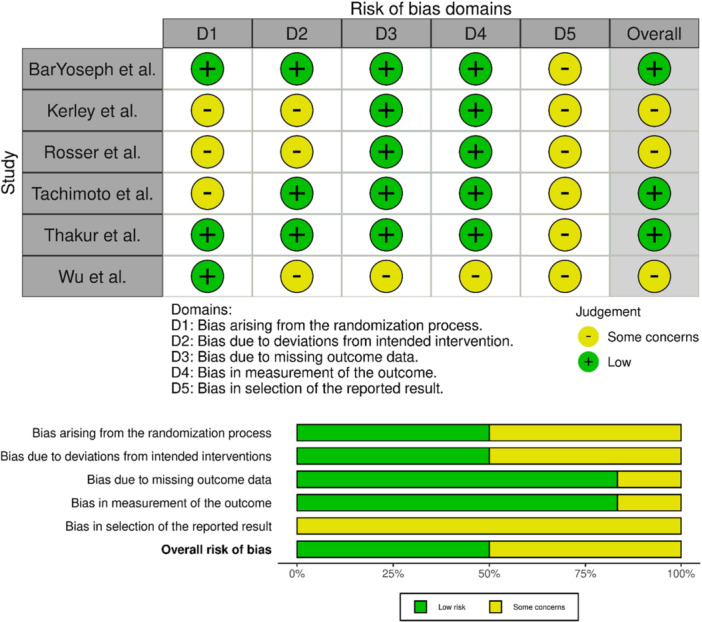
Risk of bias randomized controlled trials studies. [Color figure can be viewed at wileyonlinelibrary.com]

The assessment of the risk of bias showed that most studies were categorized as low risk or moderate quality, with the exception of the studies by Pervaiz et al. [48] and Suprun et al. [33], both of which were classified as having a high risk of bias (Tables 2, 3 and 4) (Figure 2).

**Table 2:** The characteristics of included studies ‐ cross section.

Author	Assessment of asthma control	Assessment of asthma severity	Vitamin D level and lung function	Vitamin D level and asthma control	Vitamin D level and asthma severity	Vitamin D level and IgE	Vitamin D level and treg	Vitamin D level and cytokines	Outcome	Risk of bies (JBI)
Aldubi et al. [19]	Childhood Asthma Control Test(C‐ACT)	GINA guideline 2011	positivecorrelation with PEF (*r* = 0.962, *p* < 0.0001)	positivecorrelation (*r* = 0.956, *p* < 0.0001)	negative correlation(r = −0.756, *p* < 0.0001)	no correlation	NR	positively associated with IL10 (r = 0.868, *p* < 0.0001) and an inverse correlation with TNF‐α (r = 0.313, *p* < 0.05)serum levels	Hypovitaminosis D was associated with poorer asthma control, increased exacerbations, worsened daytime symptoms, activity limitations, and higher inhaler use. FEF was a strong determinant of circulating vitamin D. No significant association was found with eosinophil counts or IgE, while an inverse correlation was observed with TNF‐α.	low risk
Amorim et al. [20]	GINA	NR	NR	NR	NR	Across the sample, vitamin D levels were moderately and significantly correlated with age and eosinophil count, but not with IgE levels. No other significant correlations were found.	NR	positively associated with IL10 (r = 0.868, *p* < 0.0001) and an inverse correlation with TNF‐α (r = 0.313, *p* < 0.05)serum levels	Vitamin D associated with eosinophil count and age in asthmatic children; causality cannot be established. International reference values may not apply to Brazilian pediatric asthma population.	Moderate risk
Asseri et al. [21]	GINA 2018	GINA 2018	NR	NR	A higher rate of vitamin D insufficiency/deficiency was observed in moderate/severe and uncontrolled asthma, but without statistical significance.	NR	NR	NR	No statistically significant differences in vitamin D status among asthma severity subgroups; a non‐significant trend was observed between vitamin D category and asthma control (*p* = 0.099).	low to moderate risk
Arikoglu et al. [22]	GINA guideline (2011)	GINA guideline(2011)	No correlation	positivecorrelation	significantly related to the riskof asthma attacks in logistic regression analysis (*p* < 0.001)	NR	NR	NR	Mean serum vitamin D levels were significantly lower in the exacerbated asthma group compared to the controlled group.	low risk
Aziz et al. [23]	GINA	GINA	NR	NR	Asthma exacerbations were significantly higher in the vitamin D deficiency group	NR	NR	NR	This study supports the hypothesis of lower vitamin D levels contributing to a higher rate of asthmaexacerbations in adolescents.	low risk
Brehm et al. [24]	Spirometry and modified versions of questionnaires used in the Collaborative Study on the Genetics of Asthma and the International Study of Asthma and Allergies in Childhood (ISAAC).	NR	No significant	NR	There was asignificant difference between the vitamin D levels in children and classification of asthma severity	inversely associated	NR	NR	Increased hospitalization risk, higher bronchodilator responsiveness, and elevated eosinophil/IgE counts correlated with lower vitamin D levels.	low risk
Brehm et al. [25]	Spirometry and modified versions of questionnaires used in the Collaborative Study on the Genetics of Asthma and the International Study of Asthma and Allergies in Childhood (ISAAC).	NR	significantly associated with FEV1/FVC (a marker of increased diseaseseverity but not with FEV1)	NR	observed association between vitamin D insufficiency and severe asthma exacerbations was greater in those who were nonatopic than in those who were atopic	no correlation	NR	NR	Vitamin D insufficiency associated with increased asthma morbidity (hospitalizations, systemic steroid use). Vitamin D insufficiency linked to 2.6 x higher odds of severe asthma exacerbation (adjusted). Insufficiency associated with lower VEF1/FVC or positive allergen‐specific IgE (adjusted).	low risk
Chinellato et al. [26]	(GINA) guidelines 2008, and Childhood Asthma Control Test (C‐ACT)questionnaire	NR	A significant positive correlation was found between FVC percent predicted, FEV 1 and serum 25(OH)D (r = 0.25, *p* = 0.049; r = 0.16, *p* = 0.16). No correlation between FEV1/FVC	A positive correlation was found between 25(OH)D and the Childhood Asthma Control Test (r = 0.28; *p* = 0.011).	NR	No statistically significant differences	NR	NR	Higher serum vitamin D correlated with better asthma control (C‐ACT), was greater in controlled vs partially controlled patients, moderately associated with FVC%, weakly with FEV1%, and showed no association with FEV1/FVC.	low risk
Dabbah et al. [27]	GINA guideline (2014)	NR	No correlation	NR	NR	NR	NR	NR	No correlation was found between serum vitamin D and FEV1, PC20, FeNO, eosinophil counts, IgE levels, or overall lung function.	low risk
Han et al. [28]	NR	NR	No correlation	NR	NR	NR	NR	Vitamin D < 30 ng/ml was significantly associated with higher IL‐5 and IL‐13 plasma levels in children with asthma	IL‐5 and IL‐13 were associated with poorer lung function and elevated IgE and eosinophils in children with asthma, particularly in those with atopic asthma. Vitamin D insufficiency modified the effect of IL‐5 and IL‐13 on total IgE and eosinophils, potentially regulating TH2 responses.	high risk
Kaur et al. [29]	GINA	GINA 2020	NR	Significantly decreased serum vitamin D3 levels in children with uncontrolled asthma compared to well‐controlled asthma.	Children with low Vit D had ↑ acute asthma exacerbations versus sufficient/insufficient groups	NR	NR	NR	This study has concluded that children with low serum levels of vitamin D₃ experienced a higher number of acute exacerbations of bronchial asthma compared to children with sufficient and insufficient levels of vitamin D₃ and had more episodes of acute bronchial asthma exacerbation compared to those with sufficient and insufficient vitamin D₃ levels.	moderate risk
Krobtrakulchai et al. [30]	GINA guideline	GINA guideline	No correlation	No correlation	No correlation	NR	NR	NR	No significant association was found between vitamin D levels and asthma control or severity.	low risk
Kuti et al. [31]	GINA guideline	National Asthma EducationPrevention Program Expert Panel Report (NAEPP)	NR	No correlation	No correlation	NR	NR	positive correlation between serum vitamin D and cytokines IL‐2, IL‐6, IL‐8; IL‐3, IL‐5, and negative correlation with IL‐13 and IL‐4	1. Lower vitamin D levels were observed in the intermittent asthma group not receiving corticosteroids compared to the non‐asthmatic children.	low risk
									2. No association was found between symptom control and vitamin D levels.	
									3. No association was found between asthma severity and vitamin D levels.	
Omole et al. [32]	GINA guideline	NR	NR	It was not statistically significant	was not statistically significant	NR	NR	NR	Children with asthma had marginally but significantly lower mean serum vitamin D levels when compared with their counterparts without asthma. However, serum vitamin D level does not seem to be associated with childhood asthma severity and control in these children with normal serum vitamin D levels.	high risk
Suprun et al. [33]	GINA 2020	GINA 2020	NR	Children with uncontrolled asthma showed more than two times lower serum vitamin D levels compared to those with controlled asthma.	NR	NR	NR	it was revealedthat children suffering fromasthma with vitamin D deficiency have truly3 times higher levels of interleukin‐5(0.62 pg/ml *vs.* 0.22 pg/ml), whichdirectly activates atopic inflammation, but at the same time significantly (4.5times) lower levels of interleukin‐4(0.2 pg/ml *vs.* 0.94 pg/ml).	1. The studies revealed that children with controlled BA have significantly higher levels of vitamin D than patients with uncontrolled disease.	high risk
									2. When studying some indicators of immune status, it was revealed that children suffering from BA with vitamin D deficiency have higher levels of interleukin‐5, but at the same time significantly lower levels of interleukin‐4.	
									3. A significantly lower proportion of active B‐lymphocytes and their absolute number were also detected. Patients with vitamin D deficiency required a significantly higher dose of glucocorticosteroids to achieve control over the disease	
Srivastava et al. [34]	GINA guideline (2021)	NR	NR	Vitamin D3 was significantly low in uncontrolled group at 9.32 ± 5.95 ng/ml, versus 12.99 ± 4.97 ng/ml in the partly‐controlled, and 13.40 ± 5.92 ng/ml in the well‐controlled group (*p* 0.005)	NR	negative correlation	NR	NR	Vitamin D deficiency was associated with elevated levels of IgE and high‐sensitivity C‐reactive protein (hs‐CRP), both of which are markers of eosinophilic inflammation. Vitamin D₃ levels were markedly low in asthmatic children and significantly lower in the uncontrolled asthma group.	low risk
Szentpetery et al. [35]	NR	severe asthma exacerbation was defined as 1 or more hospitalization, or 1 or more visit to the emergency department or urgent care for asthma that led to treatment with systemic (oral, intramuscular, or intravenous) corticosteroids, or 1 or more course of systemic steroid for asthma	NR	NR	significantly associated with non‐atopic asthma. Non‐significantly associated with severe asthma with atopic asthma.	NR	NR	Vitamin D was significantly and positively associated with IL‐10 in all subjects and vitamin D was positively associated with IL‐21, IL‐25 and IL‐31	Vitamin D deficiency was significantly associated with an increased risk of severe exacerbations among children with non‐atopic asthma but was not significantly associated with severe asthma exacerbations or hospitalizations in children with atopic asthma. Vitamin D may enhance corticosteroid responsiveness, as evidenced by a positive association between vitamin D levels and IL‐10 across all participants.	moderate risk
Tanega‐Aliling et al. [36]	NR	GINA guidelines (2011)	NR	NR	There was a significant difference between the vitamin D levels in children and classification of asthma severity	NR	NR	Serum IL‐17A levels were undetectable in 96% of the study population.	The mean serum vitamin D levels do not differ between children with asthma and healthy controls. There was no significant relationship between mean vitamin D levels and asthma severity. There was no association between the prevalence of vitamin D insufficiency and/or deficiency and asthma and its severity.	moderate risk
Uysalol et al. [37]	GINA guideline	GINA guideline	NR	negative correlation	negative correlation	NR	NR	NR	Vitamin D levels are lower in asthmatic children compared to healthy controls. Children with low vitamin D levels experienced a higher frequency of asthma attacks, more severe exacerbations, and greater difficulty in achieving asthma control.	low risk

Abbreviations: ACT, Asthma Control Test; C‐ACT, Childhood Asthma Control Test; FCV, forced vital capacity; FEV1, forced expiratory volume in one second; GINA, Global Initiative for Asthma; MCR, Medical Research Council; NAEPP, National Asthma Education and Prevention Program; NR, not reported; r, Pearson's correlation coefficient.

**Table 3:** The characteristics of included studies – cohort and case control.

Author	Assessment of Asthma Control	Assessment of Asthma Severity	Vitamin D Level and Lung Function	Vitamin D Level and Asthma Control	Vitamin D level and Asthma Severity	Vitamin D Level and IgE	Vitamin D Level and Treg	Vitamin D Level and Cytokines	Outcome	Risk of Bies (JBI)
Malheiro et al. [5]	ACT	GINA 2020	Vitamin D was positively correlated with FEV1	No differences were found in mean vitamin D levels between patients with controlled and uncontrolled asthma.	the severe asthma group had lower mean Vitamin D than the mild/moderate asthma group for both assessments	NR	NR	NR	In a tropical climate zone, there is no evidence of association between seasonality and serum Vit. D levels or between serum Vit. D levels and asthma control in children and adolescents. However, Vit. D and lung function were positively correlated and the group with Vit D insufficiency had a higher prevalence of severe asthma.	low
Batmaz et al. [38]	ACT	NR	positively correlated with FEV1 (%) (*p* = 0.031), and negatively correlated with Bronchodilator response (*p* = 0.038)	positively correlated with ACT score (*p* < 0.001)	NR	negatively correlated with Total IgE[IU/ml] (*p* = 0.041)	positively correlated with Absolut Treg count [cells/μl] (*p* = 0.034)	negatively correlated withIL‐2[IU/ml] (*p* = 0.072), IL‐4 [pg/ml] (*p* = 0.001), IL‐10 [pg/ml] (*p* = 0.283), IL‐12 [pg/ml] (*p* = 0.099), IL‐13 [pg/ml] (*p* = 0.164), TGF‐β [ng/ml] (*p* = 0.064), IFN‐γ [IU/ml] (*p* = 0.064)	Serum vitamin D levels significantly affected asthma control, Lung Function Tests, and IgE levels, independent of age, BMI, Inhaled Corticosteroids use, and season.	moderate quality
Wu et al. [39]	NR	NR	NR	NR	NR	NR	NR	NR	In children with asthma treated with inhaled corticosteroids, vitamin D deficiency is associated with poorer lung functionthan in children with vitamin D insufficiency or sufficiency	low
Chary et al. [3]	NR	NR	NR	NR	NR	NR	The 25(OH)D3 concentrations and proportion of Treg cells were lower (*p* < 0.05) among children with asthma	NR	Altered Treg cell population was directly associated with low serum vitamin D levels.	low
Abushouk et al. [40]	modified version of the GINA 2015	NR	NR	Vitamin D among patients with uncontrolled andwell‐controlled asthma showed no significant association (*p* > 0.05)	NR	NR	NR	NR	No significant difference in vitamin D levels between asthma patients and controls.	moderate quality
Bai et al. [41]	ADL (activity of daily living)	Medical Research Council‐ Dyspnea Scale (MRC)	Pearson correlation analysis showed that25OHD3 levels were positively correlated with pulmonaryfunction indexes (FEV1%pred *p* < 0.001, FVC *p* < 0.001, FEV1/FVC‐*p* < 0.001, PEF *p* < 0.001)	Pearson correlation analysis showed that25OHD3 levels were positively correlated with ADL (*p* < 0.001)	Pearson correlation analysis showed that25OHD3 levels were negatively correlated with MRC (*p* < 0.05)	NR	NR	NR	Elevated serum Vitamin A and 25(OH)D3 levels correlated with better lung function and higher Quality of Life (QoL). Lower 25(OH)D3 levels observed in asthmatic patients compared to healthy controls. Serum Vitamin A/25(OH)D3 levels and lung function indices decreased proportionally with the increasing severity of stable asthma	moderate quality
Dogru et al. [42]	NR	GINA guideline (2008)	NR	NR	The mean serum 25(OH)D level was negatively correlated with the severity of asthma (*p* = 0.001),	NR	NR	NR	Vitamin D levels were not significantly different among patients with asthma. Vitamin D deficiency was prevalent in both the study group and the control group. Clinical disease severity, frequency of exacerbations, and systemic glucocorticoid requirement were associated with vitamin D levels.	moderate quality
Maalmi et al. [43]	GINA guideline (2008) and spirometry	GINA guideline (2008) and spirometry	Serum levels of 25(OH)D were slightly associated withforced vital capacity (FVC) percent predicted(r = 0.358; *p* = 0.025). Non‐significant correlation was observedbetween percentage of forced expiratory volume in 1 s(FEV1) predicted and serum levels of vitamin D(r = 0.319; *p* = 0.0507). No correlation was observed between FEV1/FVC	NR	Patients with moderate asthmaexpressed lower vitamin D levels (14.67 ± 3.20 ng/mL)than mild asthmatics did (26.10 ± 5.85 ng/mL; *p* = 0.0001)	NR	A significant positive correlation was observed betweenserum 25(OH)D levels and the IL‐10 + CD4 + T cells(r = 0.428; *p* = 0.0081).A significant correlation was observed between the percentage of CD25highFoxp3+Treg cells and vitamin D values in asthmatics(r = 0.368; *p* = 0.021)	There was no correlation between serumconcentrations of IL‐6 and vitaminD levels(r = −0.0687; *p* = 0.677). A significant negative correlation was observed betweenIL‐17 and vitamin D levels in young asthmatics(r = −0.617; *p* = 0.001)A significant positive correlation was observedbetween vitamin D and IL‐10 levels in asthmatics (r = 0.428; *p* = 0.008).	Patients with moderate asthma (including severe asthma due to the small proportion of this subgroup) had lower vitamin D levels compared to those with mild asthma. Low vitamin D levels are associated with increased inflammatory mediators. Low vitamin D levels impair asthma control.	moderate quality
Ismail at al [44]	GINA guideline (2005)	GINA guideline(2005)	NR	significant decrease in vitamin D in clinically uncontrolled asthma (*p* < 0.001)	There was a significant decrease in vitamin D serum levels with the increase in asthma severity (*p* = 0.008)	NR	There was a significant decrease in serum vitamin D levels and CD4 + CD25+highTreg cells (%) in asthmatic group compared to the control group (*p* = 0.008). But there was no difference in the percentage of Treg (CD4 + CD25+ high Foxp3 +) cells in relation to asthma severity or clinical control	NR	Lower vitamin D levels were observed in asthmatic children compared to the control group. Vitamin D deficiency was correlated with impaired pulmonary function and associated with elevated markers of asthma severity. Vitamin D levels were lower in children with clinically uncontrolled asthma compared to those with controlled or partially controlled asthma.	moderate quality
Kalick et al. [45]	NR	AsthmaControl Test (ACT), spirometry and nitric oxide contentin the exhaled air (FeNO)	NR	no correlation	no correlation	no correlation	no correlations apparent betweenvitamin D level, and the proportion of naturalregulatory T‐lymphocytes	NR	Lower vitamin D in asthmatic children vs controls; no influence on asthma course; no relation with Treg lymphocytes.	moderate quality
Kalmarzi et al. [46]	history and physical examination +pulmonary function test	GINA guideline	positivecorrelation between the levels of vitamin D withFEV1, FVC, and FEV1/FVC(r = 0.356, 0.399,0.367)	NR	negative correlation (*r* = −0.103)	negative correlation (*r* = −0.564)	NR	NR	A significant difference in serum vitamin D levels was observed between case and control groups, with severe vitamin D deficiency predominantly associated with the case group. A significant positive correlation was found between vitamin D deficiency and pulmonary function test parameters, including FEV₁, FVC, and the FEV₁/FVC ratio. An inverse relationship was observed between serum IgE levels and vitamin D concentrations.	moderate quality
Mohammadzadeh et al. [47]	NR	Pediatric Asthma Severity Score (PASS)	NR	NR	negative correlation(*r* = −0.285, *p* = 0.004)	a significant inversecorrelation between the serum levels of 25‐OH vitaminD3 and IgE (*r* = −0.483, *p* = 0.001)	NR	NR	Patients with vitamin D3 deficiency had higher levelsof serum IgE compared with patients with insufficientand sufficient levels of vitamin D3, and Vitamin D3 deficiency or insufficiency is prevalentamong children with asthma.	moderate quality
Pervaiz et al. [48]	GINA guideline	NR	NR	NR	NR	NR	NR	there was a significantly negative correlation between Vit. D levels and IL‐17 (*r* = −6.28, *p* = 0.001). weak inverse correlation between the serum Vit. D and IL6 (*r* = −0.076, *p* = 0.61).	Vitamin D levels were significantly deficient in asthmatic patients compared to the control group; 56.6% versus 3.33%, respectively. A significant correlation was observed between vitamin D levels and inflammatory mediators in asthmatic patients.	High quality
Singh et al. [49]	NR	NR	A significant negative association was found between Vitamin D3 and FEV1/FVC in both univariate and multivariate analyses.	vitamin D3 were significantly lower in asthmatic	NR	NR	NR	NR	The study found that children with asthma had significantly lower levels of essential minerals(iron and magnesium) and vitamin D3. These elements increase with asthma risk and severity, could contribute to the disease's onset and progression in children.	low
Wang et al. [50]	NR	GINA guideline	positive correlation FEV 1 (R = 0.763, *p* < 0.05); PEF (R = 618, *p* = < 0.05);	NR	NR	NR	NR	negatively associated with the levels of inflammatory factors TNF‐α and IL‐6 (*p* < 0.05)	Eosinophil counts were lower in patients with high vitamin D levels compared to those with low vitamin D levels. Vitamin D levels showed a positive correlation with pulmonary function indices, whereas TNF‐α and IL‐6 were negatively associated with pulmonary function. Vitamin D levels in asthmatic children were negatively associated with TNF‐α and IL‐6 concentrations. Vitamin D levels in asthmatic children were lower than in health controls.	moderate quality
Wawrzynia et al. [51]	NR	AsthmaControl Test (ACT), spirometry and nitric oxide contentin the exhaled air (FeNO)	no correlation	NR	no correlation	NR	There was no significant correlation between VitaminD serum concentration and percentage of nTreg	NR	The lowest vitamin D levels were observed in children with asthma. No effect of vitamin D on asthma severity was demonstrated.	moderate quality

Abbreviations: ACT, Asthma Control Test; C‐ACT, Childhood Asthma Control Test; FCV, forced vital capacity; FEV1, forced expiratory volume in one second; GINA, Global Initiative for Asthma; MCR, Medical Research Council; NAEPP, National Asthma Education and Prevention Program; NR, not reported; r, Pearson's correlation coefficient.

**Table 4:** The characteristics of included studies–RCT.

Author	Assessment of Asthma Control	Assessment of Asthma Severity	Vitamin D Level and Lung Function	Vitamin D Level and Asthma Control	Vitamin D level and Asthma Severity	Vitamin D Level and IgE	Vitamin D Level and Treg	Vitamin D Level and Cytokines	Outcome	Risk of Bies (JBI)
Bar et al. [52]	NR	NR	No difference was found between groups regarding the number of patients achieving FEV1 improvement.	NR	NR	Despite the increase in Vitamin D, no change was observed in eosinophils and IgE.	NR	Increase in IL5, IL10, and ɣ interferon and decrease in IL17 but similar changes were observed in the placebo group	In small cohort of children with mild clinical asthma, no significant differences were observed between vitamin D monotherapy and placebo across multiple parameters, including IgE, airway cytokines, and eosinophil counts.	Low
Kerley et al. [53]	GINA 2011	c‐ACT, ACT, GINA	PL group showed non‐significant, slight advantages over the Vitamin D3 group regarding lung function, particularly FEV1%.	there were non‐significant, advantageous changes in the PL group compared with the vitamin D3 group in subjective asthma control	NR	No changes were observed in absolute IgE or eosinophil counts.	NR	NR	No significant differences were observed in C‐ACT, mPAQLQ, or GINA scores.	Some concerns
Rosser et al. [54]	NR	NR	NR	NR	NR	In a multivariable analysis, vitamin D3 supplementation had no significant effect on change in total IgE	NR	NR	In this analysis of data from a randomized, multicenter, double‐blind, placebo‐controlled clinical trial of vitamin D supplementation aimed at preventing severe asthma exacerbations in children with persistent asthma and serum vitamin D levels below 30 ng/mL, vitamin D supplementation had no significant effect on total IgE levels.	Some concerns
Tachimoto et al. [55]	GINA 2016/ACT‐cACT	NR	Vitamin D significantly reduced (at 6 months) the proportion of patients with PEF < 80 predicted	Asthma control was significantly improved in the vitamin D group compared with the placebo group.	NR	NR	NR	NR	This randomized controlled trial demonstrated a significant improvement in asthma control levels, as defined by GINA criteria and assessed using the Childhood Asthma Control Test (CACT). Although total IgE levels were higher in the vitamin D group compared to the placebo group, the differences did not reach statistical significance. Furthermore, even after adjusting for total IgE levels, asthma control remained significantly better in the vitamin D group than in the placebo group.	Low
Thakur et al. [56]	ACT/cACT	ACT/cACT	FEV1 did not differ significantly between the intervention groups.	Both groups (Vitamin D and placebo) showed improved asthma control at 12 weeks post‐intervention compared to baseline, irrespective of the intervention type.	There was no significant difference between the groups regarding the number of exacerbations, emergency visits, hospital admissions, or adverse outcomes.	NR	NR	NR	No significant improvement in asthma control, as assessed by the C‐ACT score after 12 weeks, was observed. Vitamin D supplementation did not have a significant beneficial effect on secondary outcomes, including pulmonary function tests, fractional exhaled nitric oxide (FeNO), number of asthma exacerbations, emergency department visits, and use of systemic corticosteroids.	Low
Wu et al. [57]	c‐ACT	NR	Combination therapy was superior to monotherapy, yielding higher levels of key lung function parameters (FEV1, FVC, FEV1/FVC, PEF).	Post‐treatment scores (C‐ACT and all PAQLQ domains) were significantly superior to pre‐treatment scores in both SDT and TDT groups	NR	NR	NR	The post‐treatment serum levels of IL‐6 and TNF‐α in both groups were notably inferior to the pre‐treatment levels, while the IL‐10 level was superior to the pre‐treatment level (*p* < 0.05). Moreover, after treatment, the TDT group exhibited lower serum levels of IL‐6 and TNF‐α compared to the pre‐treatment levels, while the IL‐10 level was superior to the pre‐treatment level (*p* < 0.05).	Combined treatment with fluticasone propionate inhaler and vitamin D in children with bronchial asthma may increase serum 25‐(OH)‐D3 levels, effectively modulate immunoglobulin levels and T lymphocyte subsets, control clinical symptoms, inhibit the release of pro‐inflammatory factors, and promote the expression of anti‐inflammatory mediators.	Some concerns

Abbreviations: ACT, Asthma Control Test, C‐ACT; Childhood Asthma Control Test; Pearson's correlation coefficient; FCV, forced vital capacity; FEV1, forced expiratory volume in one second; GINA, Global Initiative For Asthma; NR, not reported; PAQLQ, Pediatric Asthma Quality of Life Questionnaire; PEF, Peak expiratory flow.

### 3.2 Narrative Synthesis

#### 3.2.1 Asthma Control and Severity Assessments

Across the studies included in the systematic review, asthma control and severity were evaluated using heterogeneous tools, including GINA [58] guidelines, the Asthma Control Test (ACT) [59], spirometry, FeNO measurements, and validated clinical scores such as the MRC dyspnea scale [60] and the Pediatric Asthma Severity Score (PASS) [61]. Despite this methodological variability, a consistent trend emerged: lower serum vitamin D levels were generally observed among children and adolescents with poorer asthma control and greater clinical severity. These findings, qualitatively observed in several studies, were further supported by the quantitative results of the meta‐analyses. Variations in diagnostic criteria and assessment methods likely contributed to the substantial heterogeneity observed in the pooled estimates (Tables 2, 3 and 4).

#### 3.2.2 Immunological Markers Not Included in the Meta‐Analysis

Several studies examined the relationship between vitamin D and immunological markers that could not be meta‐analyzed due to insufficient quantitative data.

Specifically, some studies [3, 38, 43, 44, 45, 51], explored associations between vitamin D levels and regulatory T cells (Tregs), reporting inconsistent results: while a few studies described positive correlations, others found no significant associations (Tables 2, 3 and 4).

Additionally, multiple studies [19, 20, 28, 31, 33, 35, 36, 38, 43, 48, 50, 52, 57] investigated the link between vitamin D and inflammatory cytokines. Overall, lower vitamin D levels tended to be associated with changes in the Th2 inflammatory response, including higher IL‐5, IL‐13, and TNF‐α concentrations, as well as negative correlations with IL‐4 and IL‐17. Some studies also reported positive associations between vitamin D and IL‐10, suggesting potential immunomodulatory effects. However, these findings were heterogeneous and based on small samples, limiting their comparability and precluding quantitative synthesis (Tables 2, 3 and 4).

### 3.3 Main Meta‐Analysis Results

**Figure 3:**
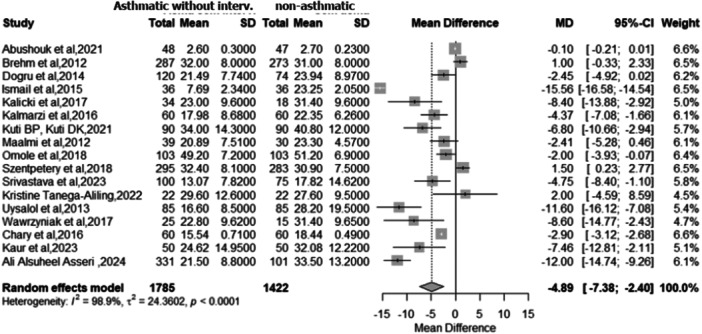
Meta‐analysis of mean vitamin D levels: asthmatic individuals without intervention versus non‐asthmatic.

The primary meta‐analysis included 17 studies [3, 21, 25, 29, 31, 32, 34, 35, 36, 37, 40, 42, 43, 44, 45, 46, 51] encompassing 3,207 participants (1,785 with asthma and 1,422 controls) out of 25 studies eligible for qualitative synthesis. Pooled analysis using a random‐effects model demonstrated significantly lower serum 25(OH)D levels in children and adolescents with asthma compared with controls with a mean difference (MD) of −4.89 ng/mL (95% CI: −7.38 to −2.40; *p* = 0.0001). High heterogeneity was observed across studies (I² = 98.9%; Q = 1479.86; *p* < 0.0001) (Figure 3).

These results highlight the potential clinical relevance of vitamin D status in pediatric asthma, suggesting that deficiency may contribute to disease risk or severity.

### 3.4 Subgroup Meta‐Analysis

Additional meta‐analyses were conducted to assess differences in circulating vitamin D concentrations according to asthma control status and disease severity (mild, moderate, severe). Furthermore, correlations between serum 25(OH)D levels and pulmonary function parameters (FEV₁ and FEV₁/FVC ratios), as well as between vitamin D concentrations and inflammatory biomarkers (total IgE, eosinophil counts, and interleukin‐10), were systematically evaluated.

#### 3.4.1 Asthma Control

**Figure 4:**
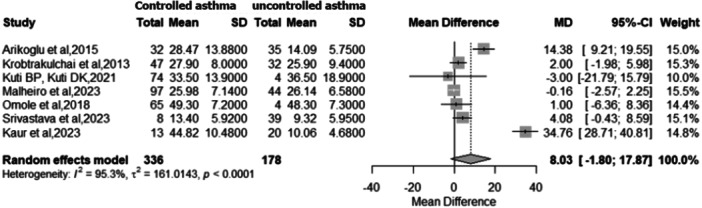
Meta‐analysis of mean vitamin D levels: Controlled asthma versus uncontrolled asthma.

Seven studies [5, 22, 29, 30, 31, 32, 34] (n = 514) compared serum 25(OH)D levels between participants with controlled and uncontrolled asthma. Pooled estimates revealed a non‐significant trend toward higher vitamin D levels in controlled asthma (MD = 8.03 ng/mL; 95% CI: −1.80 to 17.87; *p* = 0.11) (Figure 4). Heterogeneity was substantial (I² = 95.3%; Q = 127.21; *p* < 0.0001). These results suggest pronounced variability among studies, likely reflecting differences in definitions of asthma control, population characteristics, and geographic or seasonal factors influencing vitamin D status.

#### 3.4.2 Asthma Severity

**Figure 5:**
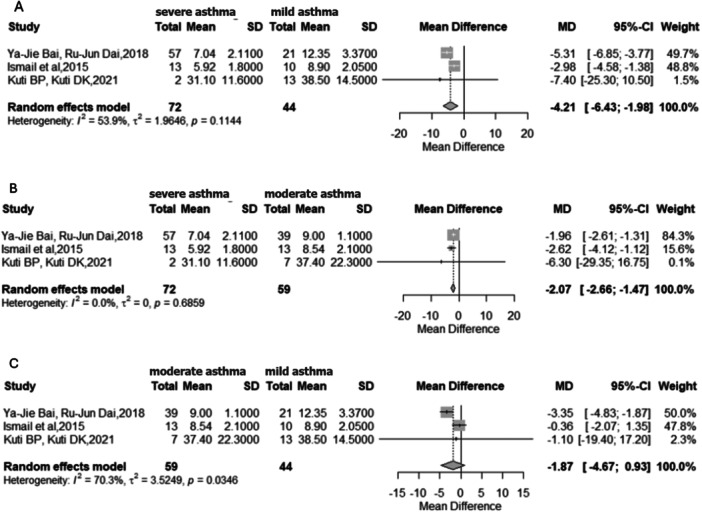
(A) Meta‐analysis of mean vitamin D levels: Severe asthma versus mild asthma. (B) Meta‐analysis of mean vitamin D levels: Severe asthma versus moderate asthma. (C) Meta‐analysis of mean vitamin D levels—Moderate asthma versus mild asthma.

In contrast, subgroup analyses [31, 41, 44] by disease severity demonstrated significant associations between serum vitamin D levels and asthma severity. Children with severe asthma had significantly lower vitamin D levels compared with those with mild disease (MD = −4.21 ng/mL; 95% CI: −6.43 to −1.98; *p* = 0.0002; I² = 53.9%) (Figure 5A). Similarly, severe asthma was associated with lower vitamin D levels than moderate asthma (MD = −2.07 ng/mL; 95% CI: −2.66 to −1.47; *p* < 0.0001; I² = 0%) (Figure 5B).

Notably, no significant differences were observed between moderate and mild (Figure 5C).

### 3.5 Correlation Meta‐Analyses

#### 3.5.1 Pulmonary Function Parameters

**Figure 6:**
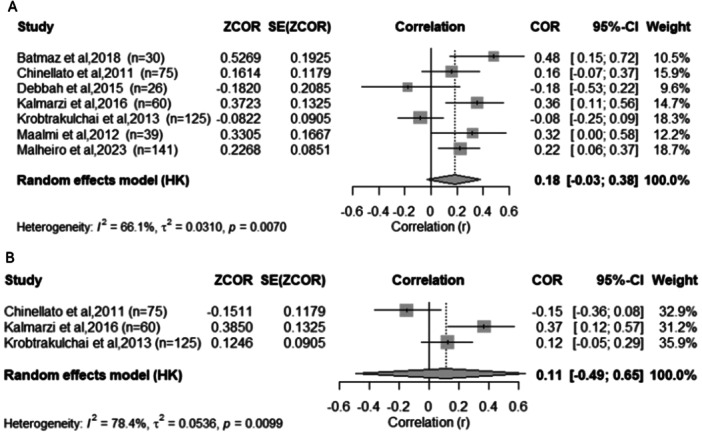
(A) Meta‐analysis of correlation data between vitamin D levels and FEV₁ (%). (B) Meta‐analysis of the correlation between vitamin D levels and FEV₁/FVC ratio (%).

Seven studies [5, 26, 27, 30, 38, 43, 46] evaluated the correlation between serum 25(OH)D concentrations and FEV₁ (% Predicted). The pooled correlation coefficient was r = 0.18 (95% CI: −0.03 to 0.38; *p* = 0.08) using a random‐effects model (Figure 6A). Heterogeneity was moderate (I² = 66.1%; Q = 17.71; *p* = 0.0070). Although not statistically significant, most studies showed a positive correlation, indicating a potential trend toward improved lung function associated with higher serum vitamin D levels. Regarding the FEV₁/FVC ratio and vitamin D, three studies [26, 30, 46] evaluated the correlation between serum 25(OH)D levels and the FEV₁/FVC ratio. The pooled correlation was r = 0.11 (95% CI: −0.49 to 0.65; *p* = 0.53), with high heterogeneity (I² = 78.4%; Q = 9.24; *p* = 0.0099) (Figure 6B). Results were inconsistent across studies, reflecting variability in sample size, asthma phenotypes, and lung function assessment methods.

#### 3.5.2 Inflammatory Biomarkers

**Figure 7:**
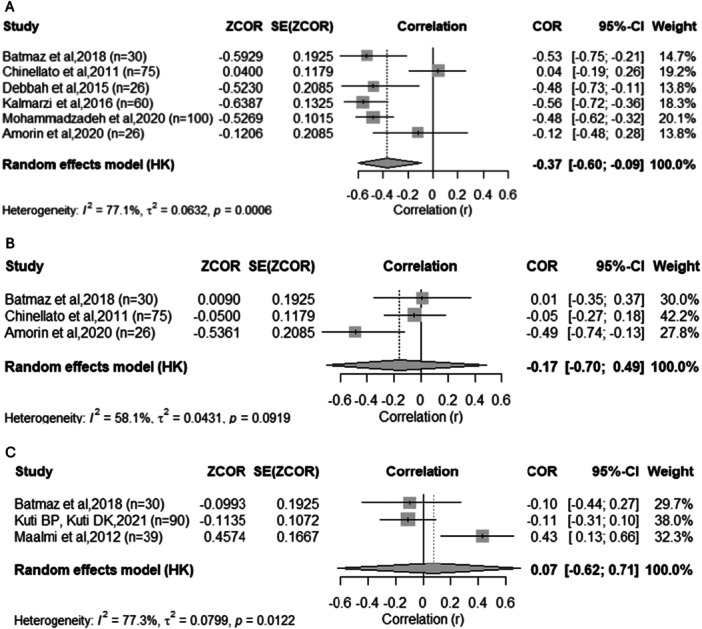
(A) Meta‐analysis of the correlation between vitamin D levels and IgE concentrations (IU/mL). (B) Meta‐analysis of the correlation between vitamin D levels and eosinophil count (cells/μL). (C) Meta‐analysis of the correlation between vitamin D levels and IL‐10 concentrations.

Six studies [20, 26, 27, 38, 46, 47] reported the correlation between serum 25(OH)D levels and total IgE concentrations. The pooled correlation coefficient indicated a moderate inverse association (r = −0.37; 95% CI: −0.60 to −0.09; *p* = 0.02) (Figure 7A). Heterogeneity was high (I² = 77.1%; Q = 21.84; *p* = 0.0006). Overall, these findings suggest that lower vitamin D levels are associated with higher total IgE concentrations in pediatric asthma.

In contrast, no significant correlations were found with eosinophil counts [20, 26, 38] (r = –0.17; *p* = 0.41) (Figure 7B) and IL‐10 levels [31, 38, 43] (r = 0.07; *p* = 0.73) (Figure 7C), suggesting that the overall implications of vitamin D with these inflammatory markers remain inconclusive.

### 3.6 Heterogeneity and Additional Analyses

High heterogeneity was observed in the primary meta‐analysis (Q = 1479.86; *p* < 0.0001; I² = 98.9%), reflecting considerable variability across studies in mean 25(OH)D levels. Comparisons between asthma control groups and severity categories also exhibited moderate to high heterogeneity (I² ranging from 53.9% to 95.3%).

In the correlation analyses, heterogeneity varied by outcome: moderate for FEV₁ (% predicted; I² = 66.1%), and high for both FEV₁/FVC ratio (I² = 78.4%) and total IgE (I² = 77.1%). These findings underscore substantial between‐study variability, likely arising from differences in populations, study designs, vitamin D measurement assays, and criteria used to classify asthma control and severity.

### 3.7 Publication Bias and Sensitivity

**Figure 8:**
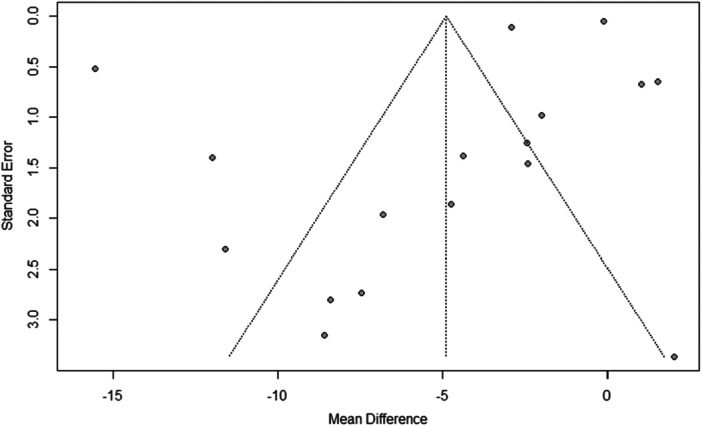
Representative funnel plots assessing publication bias.

Visual inspection of funnel plots and Egger's regression test were performed to assess potential publication bias (Figure 8). For the comparison between asthmatic and non‐asthmatic participants, Egger's test revealed no significant evidence of funnel plot asymmetry (t = −1.79; *p* = 0.093), suggesting the absence of relevant publication bias. Sensitivity analyses, conducted by sequentially excluding individual studies, did not materially alter the direction or magnitude of pooled estimates, confirming the robustness of the overall findings.

## 4 Discussion

This systematic review and meta‐analysis demonstrated that serum 25(OH)D levels are significantly lower in children and adolescents with asthma compared with healthy controls. The overall pooled mean difference of −4.89 ng/mL confirms the robustness of this association, despite substantial heterogeneity across studies. These findings indicate that vitamin D deficiency is more prevalent among pediatric patients with asthma and may be associated with a greater susceptibility to asthma attacks. Subgroup analyses further revealed that children with severe asthma had consistently lower vitamin D concentrations compared with those with mild or moderate disease. In contrast, no significant differences were observed between moderate and mild asthma. Similarly, comparisons between controlled and uncontrolled asthma showed a non‐significant trend toward higher vitamin D levels among children with well‐controlled disease. Collectively, these results suggest that low vitamin D status may be associated with poorer asthma control and greater clinical severity, although causality cannot be inferred.

In correlation analyses, higher serum vitamin D levels tended to be associated with better lung function, as reflected by a positive, though non‐significant, correlation with FEV₁ (% predicted). No statistically significant association was identified with the FEV₁/FVC ratio. Regarding inflammatory biomarkers, a moderate negative correlation was found between vitamin D and total IgE, whereas correlations with eosinophil counts and IL‐10 were not significant. Taken together, these findings suggest that vitamin D deficiency in children with asthma may contribute to enhanced allergic sensitization and immune dysregulation.

Our results were consistent with recent observational pediatric studies reporting lower 25(OH)D levels in children with more severe asthma phenotypes and, in some cases, modest positive correlations with FEV₁, though no consistent link with clinical control status was found [4]. From a global perspective, studies from Asia and Africa documented high prevalence of insufficiency/deficiency and mean 25(OH)D levels frequently below 30 ng/mL in asthmatic children. These findings support a plausible pathophysiological link and highlight the role of environmental and nutritional factors in disease expression [62].

Observational findings contrasted with evidence from randomized controlled trials (RCTs). The 2023 Cochrane update, which included both children and adults, did not find a reduction in exacerbation risk or improvement in symptom control with vitamin D supplementation, even in subgroup analyses by baseline deficiency. Notably, the representation of children with very severe asthma and marked deficiency was limited [62]. The multicenter pediatric VDKA trial conducted by Forno et al., [63] in children with baseline 25(OH)D levels below 30 ng/mL, also failed to demonstrate any benefit in preventing the first severe exacerbation with daily supplementation of 4,000 IU of cholecalciferol [63].

Conversely, more recent studies have reported mixed findings. Fedora et al. [7] conducted a meta‐analysis that demonstrated a reduction in total exacerbations with vitamin D supplementation (RR ≈ 0.62), particularly when standardized daily dosing regimens were used.7 In addition, Salameh et al. [64] reported that vitamin D may exert beneficial effects on airway structure and function by inhibiting airway smooth muscle contraction and remodeling, reducing inflammation, and regulating collagen synthesis, suggesting a potential role in mitigating airway remodeling in asthma [64]. Similarly, Hao et al. [65], in their meta‐analysis, emphasized that vitamin D supplementation may reduce lung function but does not improve asthma control. They also highlighted the need for more homogeneous, large‐scale randomized controlled trials to confirm these conclusions [65].

Regarding biomarkers, the inverse association between 25(OH)D and IgE observed in our data was consistent with a recent systematic review that estimated a negative relationship in studies with lower risk of bias [66]. However, meta‐analyses of RCTs did not demonstrate an effect of supplementation on IgE or eosinophils, although an increase in IL‑10 was reported, suggesting that cross‐sectional associations may not translate into causal effects under short‐ or medium‐term interventions [66].

Several biological mechanisms may explain the observed associations between low vitamin D levels and asthma severity. Vitamin D exerts immunomodulatory effects by downregulating cytokines such as IL‐4, IL‐5, and IL‐13, while enhancing IL‐10 production and regulatory T‐cell activity. These actions collectively suppress eosinophilic inflammation, reduce IgE synthesis, and modulate airway hyperresponsiveness [67].

Moreover, vitamin D is known to influence airway remodeling through effects on epithelial integrity, smooth muscle proliferation, and expression of inflammatory mediators, which may contribute to reduced pulmonary function in children with low vitamin D levels [67, 68].

Although these findings highlight a potential role of vitamin D in asthma pathophysiology, they should be interpreted with caution. The current evidence supports an association rather than a causal relationship. Interventional trials [7, 63, 64, 65] evaluating vitamin D supplementation in asthmatic children have shown inconsistent effects on clinical outcomes, possibly due to differences in baseline deficiency, dosing regimens, and follow‐up duration.

From a clinical standpoint, maintaining adequate vitamin D levels remains advisable for overall health, but routine supplementation specifically for asthma control cannot yet be recommended. Future studies should aim to define optimal serum thresholds for vitamin D in pediatric asthma and to clarify whether supplementation can modify disease course in deficient individuals.

### 4.1 Limitations of This Review

This meta‐analysis has several limitations. High heterogeneity across studies, particularly in the primary analysis, reflects substantial variability in population characteristics, latitude, nutritional status, assay methods, and asthma classification criteria. Most included studies were cross‐sectional, limiting causal inference. Additionally, publication bias cannot be entirely excluded despite non‐significant Egger's test results.

Strengths of this review include adherence to PRISMA guidelines, comprehensive inclusion of studies up to 2025, and separate analyses for asthma control, severity, pulmonary function, and inflammatory markers, allowing a nuanced understanding of the vitamin D asthma relationship.

## 5 Conclusion

In summary, this systematic review and meta‐analysis show that children and adolescents with asthma have significantly lower serum 25(OH)D levels compared with healthy peers, with lower concentrations associated with greater disease severity and higher total IgE levels. Although these findings support a potential immunomodulatory role of vitamin D, current evidence does not justify its use as a stand‐alone therapeutic strategy. Well‐designed randomized controlled trials are needed to determine whether correcting vitamin D deficiency can improve asthma control and long‐term respiratory outcomes in pediatric populations.

## 6 Perspectives

Future research should prioritize the inclusion of pediatric populations with moderate to severe baseline vitamin D deficiency to better assess the clinical impact of supplementation. Standardization of supplementation protocols (including dosage, formulation, and treatment duration) is essential to enable meaningful comparison across studies. Moreover, future trials should evaluate potential effect modifiers such as allergic phenotype, asthma severity, latitude and season, and exposure to air pollution.

In addition, systematic integration of type 2 inflammatory biomarkers and the use of standardized clinical outcomes consistent with current international asthma guidelines would enhance methodological rigor and improve comparability among studies.

## Author Contributions

**Joelia M. Ladeira:** conceptualization, investigation, writing – original draft, methodology, visualization, writing – review and editing, data curation. **Olívia Zacas:** conceptualization, investigation, writing – original draft, methodology. **Amanda Miranda Ferreira:** conceptualization, investigation, writing – original draft, methodology. **Patrícia Chaib Gomes Stegun:** conceptualization, investigation, writing – original draft, methodology. **Milena Baptistella Grotta:** conceptualization, investigation, writing – original draft, methodology, validation, visualization, writing – review and editing, supervision. **Adyleia A. D. Contrera Toro:** conceptualization, investigation, writing – original draft, methodology, validation, visualization, writing – review and editing, supervision.

## Funding

The authors received no specific funding for this work.

## Conflicts of Interest

The authors declare no conflicts of interest.

## Data Availability

The data that support the findings of this study are available in the References of this article.
